# Hemorrhagic Shock as Complication of Intramural Intestinal Bleeding

**DOI:** 10.1155/2017/5424631

**Published:** 2017-02-12

**Authors:** Asma Ben Ali, Mohamed Ali Cherif, Walid Mhajba, Hamdi Hamdène Doghri, Malek Hassouna, Youssef Zied El Hechmi, Zouheir Jerbi, Ines Ben Hassen, Mohamed Habib Daghfous

**Affiliations:** ^1^Emergency Department, Habib Thameur University Hospital, Faculty of Medicine of Tunis, University of Tunis El Manar, Tunis, Tunisia; ^2^Radiology Department, Habib Thameur University Hospital, Faculty of Medicine of Tunis, University of Tunis El Manar, Tunis, Tunisia

## Abstract

*Introduction*. Mural intestinal hematoma (MIH) is an uncommon complication of anticoagulant therapy. Hemorrhagic shock has been rarely reported as a revealing modality.* Results*. We report two cases of shock induced by mural intestinal hematoma in patients under oral anticoagulant for aortic prosthetic valve and atrial fibrillation. Patients were admitted to the ICU for gastrointestinal tract bleeding associated with hemodynamic instability. After resuscitation, an abdominal CT scan has confirmed the diagnosis showing an extensive hematoma. Medical treatment was sufficient and there was no need for surgery.* Conclusion*. Gastrointestinal bleeding associated with shock in patients treated by oral anticoagulant should alert physicians to research a probable MIH. Urgent diagnosis and appropriate medical treatment can avoid surgical interventions.

## 1. Case Report

**Figure 1:**
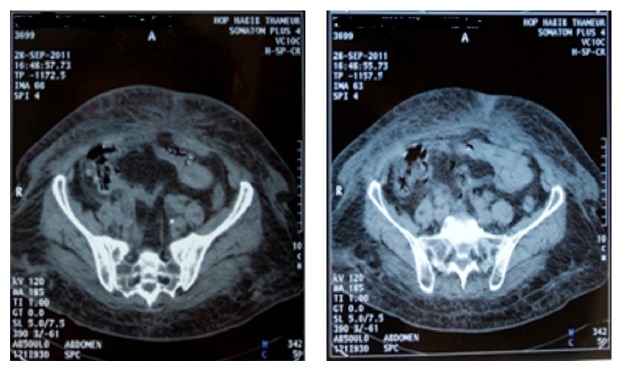
Extensive hematoma of jejunum associated with mild pelvic fluid.

*Case 1*. A 76-year-old woman was admitted to the ED for vomiting and rectal bleeding associated with an epigastric discomfort for 7 days. On initial physical examination, she was stuporous and had low blood pressure 70/50 mm Hg and a tachycardia of 100 ppm; she was marbled and had markedly pale conjunctiva and epigastric tenderness, without active rectal bleeding. She had taken oral anticoagulant for nonvalvular atrial fibrillation. She had hypertension and a labile INR. At admission, hemoglobin was 8 gr/dl, PT was over 200 sec (control 12.6 sec), and INR was so high to be measured. After resuscitation, two units of packed red cell (PRC), PPSB 30 U/kg, and vitamin K 10 mg/8 h were administered. The CT showed an extensive hematoma of jejunum associated with mild pelvic fluid (Figure 1). The pain resolved in 5 days with regression of the jejunal hematoma but persistence of pelvic liquid. Patient was discharged without oral anticoagulant.

**Figure 2:**
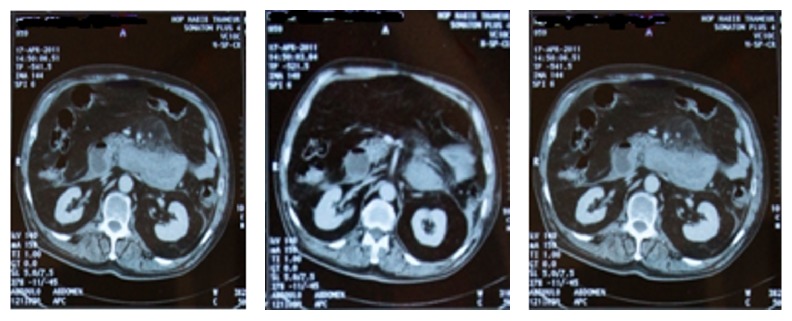
Extensive hematoma from the third portion of duodenum to the last jejunal bowel.

*Case 2*. A 69-year-old man with a prior history of hypertension, treated by oral anticoagulant for prosthetic aortic valve and atrial fibrillation, presented with a complaint of abdominal pain for 10 days and was admitted to ED for hematemesis and melena associated with intestinal obstruction syndrome. On physical examination, he was conscious and had cold extremities, low blood pressure 80/40 mmHg, and tachycardia 100 ppm; the abdomen was distended. Hemoglobin on admission was 9 g/dl. Prothrombin time (PT) was 18% with INR 6.12. Other blood tests were in normal range. After resuscitation an abdominal CT was performed, showing an extensive hematoma from the third portion of duodenum to the last jejunal bowel (Figure 2). Since his admission, oral anticoagulant was discontinued. Two units of packed red cell (PRC), vitamin K 10 mg/8h, and PPSB 30 U/kg were administered. He was discharged 15 days later with oral anticoagulant.

## 2. Discussion

We present two cases of shock induced by spontaneous mural intestinal hematoma related to excessive anticoagulation with acenocoumarol. Oral anticoagulant therapy has been reported as the most common predisposing factor [1]. The incidence of IMH among patients receiving this therapy is 1 in 2500/year [2]. The number of reported cases is increasing, probably because physicians use more often current diagnostic methods like CT scan and because of the growing number of patients under anticoagulant drugs.

The patients were male and female aged, respectively, 69 and 76 years. Oral anticoagulant was used for prosthetic valve and persistent atrial fibrillation. In a review by Sorbello et al., the incidence was higher in males, with ages between 32 to 78 years [3].

Suspicion of MIH is the first step in the diagnosis. MIH has been reported in many papers. Abdominal pain is the main symptom, and according to authors MIH should be considered in any patient with abdominal pain who is receiving long-term anticoagulation therapy. In our report, both of the patients had gastrointestinal bleeding with hemorrhagic shock. This entity was rarely reported in literature. In a review of 24 reports, gastrointestinal hemorrhage, including hematemesis, melena, or rectal bleeding, is present in less than half of the patients [3, 4] and hemodynamic instability related to massive bleeding was scarcely reported [5]. In a review of 170 cases of traumatic and spontaneous hematomas published in the literature, Birns et al. [6] reported intraluminal gastrointestinal tract bleeding in 30% of cases, with major bleeding in 3.5% of patients. We suggest that MIH should be evoked in patients presenting with gastrointestinal bleeding with hemorrhagic shock receiving a long-term anticoagulant therapy.

The INR levels of all patients were detected to be high. Both of the patients had massive bleeding and were anemic on admission. Laboratory tests usually reveal PT, PTT, and INR value above the range of normal (mean INR value 4.40–11.6) [1, 4]. However, there were reported cases of intramural hematoma in patients with PT in normal range [4] and authors show that there is no close correlation between bleeding and the level of prothrombin activity. In literature review, most patients were not anemic on admission; anemia was developed within the first 48 hours [1, 6].

Multiple scores have been established to stratify bleeding risk. ACUITY and CRUSSADE score are similar to predict major bleeding and better than HASBLED but it stills the most used one because of its simple calculation [7]. The reported cases were at a high risk of major bleeding being elderly and having hypertension and labile INR. We admit also that factors such as long time for consultation and a high INR value could explain the occurrence of major bleeding in the reported cases.

CT scan is a sensitive method, and it can be considered the gold standard of the diagnosis of MIH. To detect the hematoma, it must be done without contrast [3, 4]. Abbas et al. found that the signs are a thickening of the wall greater than 1 cm, with partial reduction to total obstruction of the passage, and the “pseudokidney” and “coiled spring” which are generally observed in short segments (average 23 cm) with profuse dilation of the flaps [1]. According to Sorbello et al. review the small bowel is mostly affected in jejunum (71,6%), followed by duodenum (29,8%) and ileum (15,8%) [3]. Most (85%) are single and nonextensive hematoma, but multiple hematoma or small bowel involvement accompanied with hematoma in the large bowel can be found. Intraluminal, intramesenteric, and retroperitoneal hemorrhage can occur with intramural hematoma [6], especially when the duodenum is involved [8].

All cases were managed medically, with discontinuing of anticoagulant drugs, correction of coagulopathy with PPSB and vitamin K, and correction of anemia. The outcomes were good and there was no need of surgical intervention. Based on the literature, PPSB with vitamin K treatment can correct coagulation parameters into normal range within 72 hours [4]. Symptoms usually improved within a few days. Complete resolution of the hematoma usually occurs within 3 weeks, varying from 10 days to 2 months [1, 5, 9].

Despite the severity in clinical presentation, there was no need of surgery in the reported cases. According to authors, surgical treatment should be reserved for patients in the evidence of complications such as suspected ischemia with or without bowel perforation, peritonitis, intra-abdominal hemorrhage, and intestinal occlusion [10]. The factor determining prognosis is the extension of the hematoma [1]. High mortality rate was observed in patients with extensive hematoma, such as involvement of more than half the length of the small bowel.

## 3. Conclusion

MIH is an uncommon complication in patients with excessive anticoagulation. Gastrointestinal tract bleeding associated with shock was an unusual manifestation. MIH should be evoked in patients presenting with gastrointestinal bleeding associated with hemorrhagic shock who receive anticoagulation therapy. Computed Tomography is the diagnostic modality of choice. Urgent diagnosis and appropriate medical treatment can avoid unnecessary surgical interventions.
